# Growth rate, transmission mode and virulence in human pathogens

**DOI:** 10.1098/rstb.2016.0094

**Published:** 2017-03-13

**Authors:** Helen C. Leggett, Charlie K. Cornwallis, Angus Buckling, Stuart A. West

**Affiliations:** 1 Department of Genetics, University of Cambridge, Cambridge CB2 3EH, UK; 2 Department of Biosciences, University of Exeter, Cornwall Campus, Penryn TR10 9FE, UK; 3 Department of Biology, Lund University, 223 62 Lund, Sweden; 4 Department of Zoology, University of Oxford, Oxford OX1 3PS, UK

**Keywords:** transmission, virulence, parasites, growth, trade-offs, infective dose

## Abstract

The harm that pathogens cause to hosts during infection, termed virulence, varies across species from negligible to a high likelihood of rapid death. Classic theory for the evolution of virulence is based on a trade-off between pathogen growth, transmission and host survival, which predicts that higher within-host growth causes increased transmission and higher virulence. However, using data from 61 human pathogens, we found the opposite correlation to the expected positive correlation between pathogen growth rate and virulence. We found that (i) slower growing pathogens are significantly more virulent than faster growing pathogens, (ii) inhaled pathogens and pathogens that infect via skin wounds are significantly more virulent than pathogens that are ingested, but (iii) there is no correlation between symptoms of infection that aid transmission (such as diarrhoea and coughing) and virulence. Overall, our results emphasize how virulence can be influenced by mechanistic life-history details, especially transmission mode, that determine how parasites infect and exploit their hosts.

This article is part of the themed issue ‘Opening the black box: re-examining the ecology and evolution of parasite transmission’.

## 1. Introduction

There is huge variation across pathogen species in the harm they cause their hosts during an infection (virulence). Some bacteria, such as *Bacillus cereus*, cause humans mild nausea and diarrhoea for 24–48 h [1]. By contrast, other species such as *B. anthracis* kill 90–100% of their human hosts, often within 48 h [2].

Over the last 30 years, there has been a proliferation of theoretical models developed to explain this variation in virulence [3–12]. A fundamental assumption in these models is that there is a trade-off between parasite growth and host survival—a higher parasite growth rate increases parasite transmission rate, but also increases host mortality (virulence) leading to a shorter transmission period [3,7]. As a result of this trade-off, theory predicts that transmission rates will be maximized at an intermediate growth rate, with the associated levels of virulence being an unavoidable consequence. This theory has been supported by a number of within-species studies which have examined either the correlation between pathogen growth rate and virulence, or how virulence evolves in response to changing conditions such as co-infection of multiple parasite strains and host population structure [8,12–19].

By contrast, evidence that the trade-off theory explains the large differences we observe across parasite species in virulence is lacking [12,18,20–22]. One possible explanation for why patterns of virulence across species have been difficult to explain is that mechanistic interactions between hosts and parasites may vary across species and swamp any simple relationship between growth rate and virulence [10,12,18]. For example, if traits that have large fitness benefits to parasites also cause pathogenesis, for instance when the symptoms of infection aid transmission, or when parasite strategies used to escape or modulate the immune system cause mortality, then any effect of growth rate on virulence may be hidden if such traits are not considered [12]. The apparent discord between the predicted influence of growth rate on virulence and the observed cross-species patterns in virulence may, therefore, be resolved if the mechanisms via which parasite are transmitted, as well as the way parasites evade host immunity, are examined in concert with growth rate.

Here, we use comparative analyses to examine the relationship between virulence, growth, transmission and mechanisms to evade host immunity across 61 human pathogens. We first tested whether virulence was positively correlated with parasite growth rate, as commonly assumed by virulence theory. We then examined whether virulence correlated with six life-history variables that have previously been argued to influence the mechanism of pathogenesis (table 1): (i) the route of infection, which can influence the extent to which virulence decreases parasite transmission; (ii) whether symptoms of parasite infection aid parasite transmission; (iii) whether a species can kill or survive within professional immune cells (immune subversion); (iv) whether a species primarily requires transmission to humans to complete its life cycle (obligate human parasites) or not (facultative human parasites); (v) whether the production of virulence factors is coordinated cooperatively with signalling (quorum sensing); and (vi) the ability of a species to move (motility) and hence disperse between areas within a host. We also tangentially consider how the number of infectious particles required to establish an infection (infective dose) covaries with growth rate, because infective dose has previously been shown to be an important determinant of virulence [29,41].

**Table 1.** Parasite life-history variables commonly suggested to influence virulence.

parasite life-history variable	predicted effect on virulence
*route of infection* how a parasite gets exposed and transmitted to new hosts	(i) in cases where virulence does not hinder transmission, such as when vectors circumvent the need for an ambulatory host, a higher virulence can be favoured [23–27]. (ii) transmission routes which lead to higher within-host strain diversity can favour either higher [7] or lower [10] virulence.
*symptoms of infections* whether the host's symptoms of parasite infection aid parasite transmission	virulence will be higher when virulence aids transmission [23–26].
*immune subversion* whether a species can kill or survive within professional immune cells (immune subverter) or not (non-immune subverter)	investing in control of host immune system trades off with efficient host resource extraction, leading to reduced growth and virulence [28,29] or mechanisms of pathogenesis that target the immune system will be correlated with relatively high virulence because the benefits of increased parasite growth and survival will outweigh the cost of an increased host mortality [12].
*facultative* versus *obligate parasitism* whether human infection is a required part of the life cycle of the parasite (obligate) or not (facultative)	facultative species will be less adapted to infecting humans and so will cause lower virulence [30]. Observation bias could distort this trend, if relatively more virulent facultative infections were more likely to be studied.
*quorum-sensing controlled virulence factors* whether the production of virulence factors is controlled cooperatively	coordinated production of virulence factors leads to more efficient host exploitation that increases virulence by greater ‘force’ of attack [29,31] or decrease virulence by avoiding unnecessary damage [29,32,33].
*motility* the ability of a species to move (motility) and hence disperse between areas within a host	motility facilitates dispersal and colonization leading to increased virulence [34–37] or decreased virulence because motility appendages are costly to make and facilitate clearance by host immune cells [38–40].

## 2. Material and methods

### (a) Collection of data from the literature

An issue in any comparative study is that patterns in the data are highly sensitive to the choice of taxa, raising the question of whether the results observed might be due to biases in the data available. An obvious source of bias in our data collection is that the parasites for which we have the most data are the ones demanding medical intervention. Related to this is the matter of how to score a pathogen when its symptoms and life history vary widely across patients and infection routes: there is likely a bias to record the worst types of infections by pathogens, with asymptomatic infections by the same parasite going unrecorded (whether intentionally or unintentionally). Given these limitations and to ensure our analysis was as informative as possible, we restricted our dataset to only include species for which other corresponding data were available. Specifically, (i) we included only pathogens where we had *in vitro* growth rate data of human isolates, although it is unclear to what extent the strains had been ‘domesticated’ in the laboratory and (ii) where multiple transmission routes were possible, we incorporated the infection route and symptoms described in the case fatality rate data.

To minimize variation across patients, the case fatality rates are estimates of fatality without treatment or other illnesses and represent the number of cases of a disease ending in death compared with the number of cases of the disease. We classified routes of infection used by pathogens as entry through wounded skin, inhalation or ingestion. We obtained data for case fatality rate, route of infection, facultative versus obligate parasitism, symptoms of infection and the number of pathogen cells required to start an infection (infective dose) by searching (i) databases from the United States Food and Drug Administration [1], Health Canada [42] and the Center for Disease Control and Prevention [43]; and (ii) direct searches in the empirical literature using keyword searches in the ISI Web of Knowledge database, Google Scholar and PubMed.

Because *in vivo* human infection data are unavailable and data from other host species can differ, we used *in vitro* generation time as a measure of parasite growth rate, with smaller generation times implying a higher growth rate. Presumably, the *in vitro* conditions are ideal for parasite growth, and so here we are analysing ‘maximum’ growth rate, rather than the growth rate within hosts. Yet, all things being equal, we expect *in vitro* growth rate to be correlated with *in vivo* growth. In support of this, (i) *in vitro* growth measures have been shown to correlate with genomic traits associated with fast growth (such as rRNA and tRNA copy number) [44] and (ii) experimental evidence has found that *in vitro* and *in vivo* growth are correlated within species [44,45]. Data on the growth rate of pathogens (minimal generation times in hours) were collected from the electronic supplementary material of Vieira-Silva & Rocha [44] and Gama *et al.* [29] with some new data from the primary literature. Rate data can be modelled as a Poisson process once converted to counts per time. We, therefore, transformed generation times into number of generations per week for analytical purposes. The period of a week was chosen as this resulted in little rounding error when data were converted to whole number counts and is easily interpretable in a biological/clinical setting. For interactions with the immune system, motility and quorum sensing, we first followed the electronic supplementary material of Gama *et al.* [29], with new data from the primary literature which we found by searching the aforementioned databases. We have included all data and bibliographic references in electronic supplementary material, table S6.

### (b) Statistical methods

We analysed our data using multi-response Bayesian phylogenetic mixed models (MR-BPMMs) with Markov chain Monte Carlo estimation in the R package MCMCglmm, v. 2.21 [46,47]. MR-BPMMs have three main advantages in the context of this study. (i) They allow the phylogenetic variance and covariance between multiple response variables to be modelled simultaneously, which enables both the phylogenetic and residual (non-phylogenetic) correlations to be estimated while examining the effects of other potentially influential explanatory variables on all the response variables concurrently (e.g. estimates the effect of the other life-history traits in our dataset on case fatality rate, growth rate and infective dose simultaneously). (ii) In phylogenetic mixed models, explanatory variables for each species are assumed to be fixed over evolutionary time and measured without error. Including growth rate and infective dose as response variables, as well as case fatality rate, makes it possible to model the evolutionary change in these variables over time. (iii) It relaxes the assumption of causality (e.g. that growth rates predict case fatality rate) allowing for the possibility that case fatality rate may feedback and influence the evolution of growth rate.

To model the effects of phylogenetic history and account for the non-independence of data arising due to common ancestry between pathogen species, we generated a phylogeny of the species in our dataset using the Structural Classification of Proteins (SCOP) database v. 1.75 (http://supfam.org/SUPERFAMILY/cgi-bin/genome_names.cgi). SCOP constructs phylogenetic trees on the basis of structural protein similarities derived from whole genome sequencing. Where more than one genome for a species was available in the database, we selected genomes of human isolates. The branch lengths of phylogenies produced by SCOP represent divergence in protein structure and are non-ultrametric. Models fit in MCMCglmm require trees to be ultrametric where branch lengths provide some estimate of evolutionary time. We, therefore, converted the tree to be ultrametric using the ‘chronopl’ function in the R package ‘ape’ v. 3.1.2 with root-to-tip length and lambda set to 1 [48]. We modelled phylogenetic history in our MR-BPMMs by fitting a variance–covariance matrix constructed from the phylogenetic tree as a random effect where the correlation in the response trait between two pathogen species is inversely proportional to time since their most recent common ancestor, assuming a Brownian model of evolution. To assess the sensitivity of our models to branch length information, we repeated our analyses with branch lengths set arbitrarily to be equal to 1 and recovered the same qualitative results as presented in electronic supplementary material, tables S1–S5.

We conducted four sets of analyses. First, we tested whether there was a positive correlation between case fatality rate and pathogen growth using a MR-BPMM with case fatality rate (% deaths: binomial distribution) and generations per week (number per week: Poisson's distribution) as the response variables. We fitted separate intercepts for each response variable as fixed effects and estimated the phylogenetic and residual correlations between case fatality rate and generations per week by fitting 2 × 2 unstructured covariance matrices as random effects. Phylogenetic and residual correlations between traits were calculated as the posterior mode and 95% credible interval (CIs) of the posterior distribution of variance in trait A/square root (variance in trait A × variance in trait B). Correlations were considered statistically significant when the 95% CI did not span 0 and less than 5% of posterior samples were greater or less than 0 (pMCMC < 0.05) [46]. We quantified the amount of variation in response variables explained by phylogenetic history by calculating the posterior mode and CI of phylogenetic heritability (phylo-*H*^2^ = phylogenetic variance/sum(phylogenetic variance + residual variance)). The results of this analysis are presented in electronic supplementary material, table S1.

Second, as the majority of the species in our dataset are bacterial pathogens, we tested that the results in the first analysis were not driven by a few distantly related taxa by repeating the analysis only including data on bacteria. The results of this analysis are presented in electronic supplementary material, table S2.

Third, we tested how the case fatality rate and pathogen growth rate (and their correlation) were influenced by other life-history and epidemiological factors by rerunning the first analysis, but including immune subversion (two-level fixed factor: yes versus no), infection route (three-level fixed factor: ingestion, inhalation and skin), whether symptoms aid transmission (three-level fixed factor: hinder, no effect, help), pathogen motility (two-level fixed factor: yes versus no), if pathogens use quorum sensing to regulate their growth (two-level fixed factor: yes versus no) and whether humans were obligate or facultative hosts for pathogens (two-level fixed factor: yes versus no) as fixed effects. The magnitude and statistical significance of relationships between fixed effects and response traits (case fatality rate and pathogen growth) was examined using the posterior mode, 95% CI and pMCMC of fixed effects. The results of this analysis are presented in electronic supplementary material, table S3. As above, we also repeated the analysis only including data on bacteria. The results of this analysis are presented in electronic supplementary material, table S4.

Fourth, as it has previously been shown that infective dose is an important determinant of case fatality rate and may covary with pathogen growth rate [29,41], we repeated our third analysis including infective dose as a third response variable (number of pathogens required for infection: Poisson's distribution with log link function). This enabled us to calculate the phylogenetic and residual correlations between case fatality rate, pathogen growth rate and infective dose while testing for the influence of life-history and epidemiological factors on these variables and their relationships. We once again fitted separate intercepts for each of the three response variables and estimated phylogenetic and residual correlations between response traits by fitting 3 × 3 unstructured covariance matrices as random effects. The results of this analysis are presented in electronic supplementary material, table S5.

Prior to all analyses, we *Z*-transformed (mean = 0, standard deviation = 1) continuous fixed effects and converted two-level fixed effects to binary coding −1, 1 so that we could directly compare the magnitude of parameter estimates [49,50]. We ran MR-BPMMs for 10 000 000 iterations with a burn-in of 5 000 000 and a thinning interval of 5000. We checked the convergence of each analysis by manual inspection of the MCMC chains of the posterior distribution and using the Gelman–Rubin statistic (potential scale reduction factor, PSR) to compare within- and between-chain variance [51] in the R package ‘coda’ [52]. We examined the sensitivity of our results to prior specification by repeating each analysis with two different priors: an inverse-Gamma (*V* = 1, *ν* = 0.002) and parameter extended ‘Fisher’ prior (*V* = 1, *ν* = 1, *α*·*μ* = 0, *α*.*V* = 1000). The results of models were extremely similar regardless of prior specification, but the convergence of models with the parameter extended model was slightly better and so we present only results from these models. The R code used to specify priors and fit MR-BPMMs is presented in the electronic supplementary material. All parameter estimates in electronic supplementary material, tables S1–S5, are presented on the scale of the link function of models (binomial = logit, Poisson = log).

## 3. Results and discussion

### (a) Virulence and growth rate

**Figure 1.**
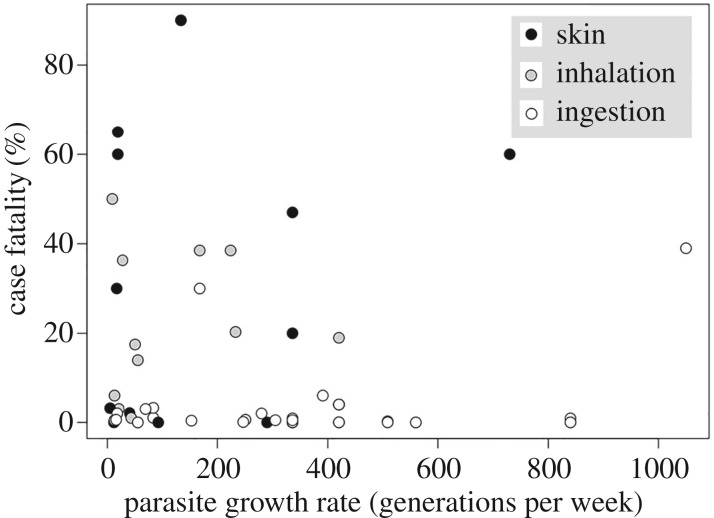
Virulence and growth rate. White circles are ingested parasites; grey circles are inhaled parasites; black circles are parasites that infect via skin wounds. Smaller generation times imply faster growth. We found a significant negative relationship between case fatality rate (%) and generation time (generations per week) (electronic supplementary material, tables S1 and S2).

Our first aim was to test whether virulence is positively correlated with parasite growth rate. In contrast with the standard assumption that increased parasite growth leads to greater host mortality [3,7], we found the opposite that there was a significant negative correlation between generation time and virulence (figure 1; electronic supplementary material, table S1, *p* = 0.009). This does not demonstrate that the trade-off theory for virulence is inaccurate or does not exist. The significant negative relationship between growth rate and virulence could occur because other life-history details that determine how parasites infect and exploit their hosts change the predicted trade-off between growth, transmission and virulence.

### (b) Virulence and transmission

**Figure 2.**
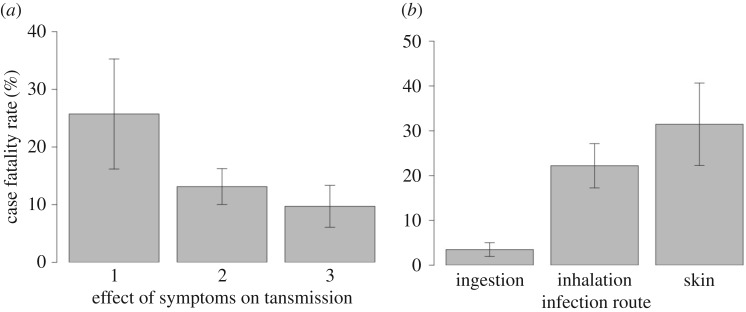
Virulence and parasite transmission. The case fatality rate (%) was: (*a*) not correlated with how symptoms of infection affect (1, hinder; 2, no effect; 3, help) transmission (electronic supplementary material, table S3) but (*b*) significantly higher in species transmitted via inhalation and wounded skin than ingestion.

Our second aim was to examine whether parasite virulence correlated with the route of infection, which can influence the extent to which virulence decreases parasite transmission between hosts for a number of reasons (table 1). For example, it has been argued that vectors can circumvent the need for ambulatory hosts for transmission and can lead to higher strain diversity, both of which could lead to higher virulence for vectored parasites [7,25,26]. We tested whether virulence was higher in parasites where virulence aids transmission, but found no significant relationship between virulence and whether the host's symptoms of infection hinder, have no effect or help transmission (figure 2*a*; electronic supplementary material, table S3, all pMCMC < 0.05). However, we found that inhaled pathogens result in significantly higher virulence than ingested pathogens (figure 2*b*; electronic supplementary material, table S3, pMCMC < 0.0001), but not pathogens infecting via skin wounds (figure 2*b*; electronic supplementary material, table S3, pMCMC = 0.30). Pathogens infecting via skin wounds are also significantly more virulent than ingested pathogens (figure 2*b*; electronic supplementary material, table S3, pMCMC < 0.0001).

This pattern is different from a previous analysis, where pathogens infecting via skin wounds were more virulent than the other two types [41]. The difference between these two studies is driven by differences in the data: we now have a larger dataset with more-virulent inhaled pathogens that were not included in the previous paper because we had no infective dose data for them. Also, the majority of our data now comprises bacteria, because there is a lack of appropriate growth rate data for viruses. To check that our results were not driven by data on under-represented groups, we conducted the same analysis with just the bacteria data that made up 82% of our dataset (*N*_species_ = 50). We obtained the same results for bacteria as we did when we analysed the entire dataset (electronic supplementary material, tables S2 and S4).

### (c) Virulence and variation in parasite infection strategies

Establishment of a successful infection is not just predicted to depend on transmission and growth potential, but also on other life-history details and epidemiological factors that determine how parasites infect and exploit their hosts. Frank & Schmid-Hempel [12] argued that mechanisms of pathogenesis that target the immune system will be correlated with relatively high virulence because the benefits of increased parasite growth and survival will outweigh the cost of increased host mortality. Whether a species is a facultative or an obligate parasite of humans could matter because facultative species might be less adapted to their hosts and hence cause lower virulence [30]. Quorum sensing could matter because it allows pathogens to produce virulence factors more efficiently [31], which could either facilitate a greater attack on the host, or avoid unnecessary damage to the host [29,32,33]. Motility can also lead to a higher virulence by facilitating dispersal and colonization, allowing pathogens to counteract mucus flows and peristalsis [34–37]. Alternatively, motility could decrease virulence because flagella mediate the activation of dendritic cells, greatly reducing bacterial survival [38], are costly to make [39] and facilitate phagocytosis [40,53].

We investigated whether parasites (i) can kill professional phagocytes or can survive and/or replicate in the intracellular milieu of these cells, termed ‘immune subverters; (ii) require (obligate parasites) or do not require (facultative parasites) a human host to complete their life cycle; (iii) use quorum sensing to regulate the production of virulence factors; and (iv) are motile within their host. Contrary to predictions, we found no significant relationship between virulence and whether a parasite species is (i) capable of subverting the immune system or not (electronic supplementary material, figure S2*a* and table S3, pMCMC = 0.27); (ii) a facultative or obligate parasite (electronic supplementary material, figure S2*b* and table S3, pMCMC = 0.23); (iii) using quorum sensing to control virulence factor expression (electronic supplementary material, figure S2*c* and table S3, pMCMC = 0.43); or (iv) motile or not (electronic supplementary material, figure S2*d* and table S3, pMCMC = 0.19).

### (d) Infective dose and virulence

**Figure 3.**
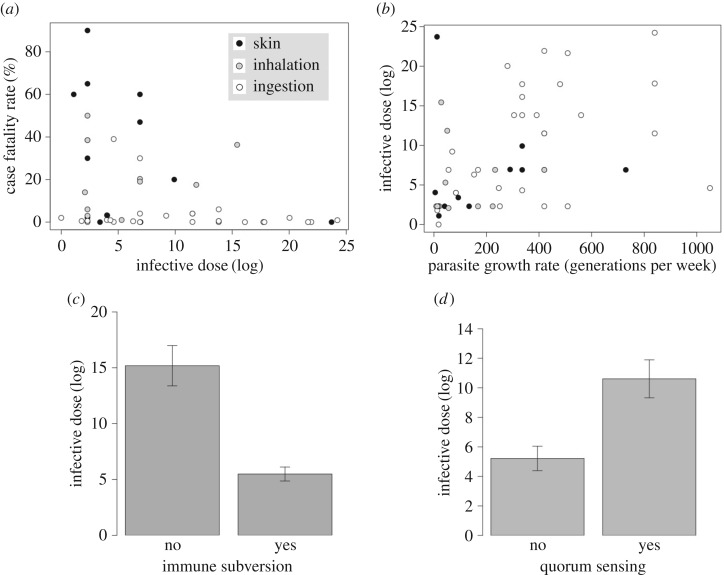
Correlates of infective dose. (*a*) The case fatality rate (virulence) was significantly higher in species with a lower infective dose (log). (*b*) The generation time (generations per week) was significantly higher in species with a higher infective dose. White circles are ingested parasites; grey circles are inhaled parasites; black circles are parasites that infect via skin wounds. (*c*) Infective dose was significantly lower in pathogens with immune subversion. (*d*) Infective dose was significantly higher in pathogens with coordinated production of virulence factors (electronic supplementary material, tables S3 and S5).

We previously found that the number of cells required to successfully infect a host (infective dose) was significantly negatively correlated with virulence [41], a pattern that we find again in this extended dataset (figure 3*a*; electronic supplementary material, table S5, pMCMC < 0.001). Our current results suggest that this is because (i) infective dose is positively correlated with parasite growth (figure 3*b*; electronic supplementary material, table S5, pMCMC = 0.02)—this is a phylogenetic correlation suggesting that infective dose and parasite growth have coevolved over evolutionary time; but (ii) faster growing parasites are less virulent (figure 1, electronic supplementary material, table S1 pMCMC = 0.0009) than slower growing parasites. Hence, higher parasite growth is correlated with higher infective dose, which is correlated with lower virulence.

Gama *et al.* [29] previously found that immune subversion was the most significant factor shaping variation in infective dose among human pathogens. Consistent with this, we find that immune subverting parasites have a significantly lower infective dose than parasites that do not subvert host immunity (figure 3*c*; electronic supplementary material, table S5, pMCMC < 0.001). This seems intuitive if we consider that, from an individual parasite's perspective, investing in control of the host immune system trades off with efficient resource extraction, leading to reduced growth [28,29]. Also, a parasite that can subvert host immune cells presumably does not need to outpace immune clearance in order to successfully establish an infection. Together these results suggest that the correlation between growth and virulence (figure 1) maybe the result of slow growing species adopting different infection strategies whereby they target the immune system causing more damage. By contrast, fast growing parasites with high infective doses do not kill or subvert professional immune cells leading to a higher probability of recovery from infection [12].

We also found that parasites that cooperatively control their production of virulence factors (quorum-sensing controlled virulence factors) have higher infective doses (figure 3*d*; electronic supplementary material, table S5, pMCMC = 0.008) and grow significantly faster (electronic supplementary material, figure S3*b* and table S5, pMCMC = 0.05) than parasites that do not. This seems logical if we consider that quorum sensing is dependent on population density and so is likely most effective when infective dose is high and/or when parasites reproduce quickly. Together with the fact that fast growth is correlated with low virulence (figure 1), this suggests that quorum-sensing parasites may be less virulent, perhaps because their coordinated production of virulence factors avoids unnecessary host damage [29,32,33].

### (e) Insights and conclusions

In general, there have been only a limited number of formal comparative studies attempting to explain virulence across a number of pathogen species [20,26,41,54–56]. An important exception is Herre's [20] work on fig wasp nematodes, which showed higher virulence in species where there was a higher chance of transmission. A key difference between our studies is that the fig wasp nematodes all shared very similar life histories, and so there were not the large mechanistic life-history details that have been a focus of our study here. Indeed, the absence of such variation is critical to Herre's study by facilitating the influence of variation in only one key variable, transmission rate. Our results indicate that when examining parasites over broader taxonomic levels that encompass different mechanisms to infect hosts, the simple predicted relationship between transmission and virulence may not be borne out.

*A priori* we might expect our correlations to give little insight into causation. However, there are at least three important reasons why a study of growth rate and virulence correlates is pertinent. First, while the typical caveats apply concerning data quality and correlation not necessarily reflecting causation, obtaining alternative experimental data is problematic because (i) it is almost impossible to conduct controlled *in vivo* infections in humans, especially when many of the pathogens of interest are classified as potential bioweapons, and (ii) interpolating results from model animal infections is complicated by ethical constraints and variations in disease patterns between different host species. Second, there is a vast theoretical literature exploring virulence evolution, with so many permutations, that it is difficult to see the wood for the trees [57]. Distilling the available data to identify broad-scale patterns could yield useful results, but it has not been done previously with respect to growth rate and virulence. It might be that a particular causal factor is so important that it overwhelms all other variables, and the correlation with that variable shines through. With infectious diseases, this would be good to know, that is, a variable that we wish to note above all others. Third, many verbal arguments about virulence evolution have been made using correlational support from a small number of pathogens, mostly different strains of the same species [23–26]. To an extent, our analysis weeds out some of these arguments, suggesting that there perhaps was/is no basis for them.

In conclusion, we found that (i) slower growing pathogens are significantly more virulent than faster growing pathogens, (ii) inhaled pathogens and pathogens that infect via skin wounds are significantly more virulent than pathogens that are ingested, but (iii) there is no correlation between symptoms of infection that aid transmission (such as diarrhoea and coughing) and virulence. The ability of comparative analyses to confirm some predicted relationships with virulence while questioning the effect of other variables highlights the utility of this approach in further understanding the evolution of virulence in different species under different epidemiological, ecological and evolutionary settings.

## Supplementary Material

ESM for growth, transmission and virulence

## Data accessibility

The datasets and analysis coding supporting this article have been uploaded as part of the electronic supplementary material.
