# Supplementary material for: Growth rate, transmission mode and virulence in human pathogens
Source: Philos Trans R Soc Lond B Biol Sci. 2017 Mar 13;372(1719):20160094. doi: 10.1098/rstb.2016.0094 (PMC5352820; doi:10.1098/rstb.2016.0094)
Supplement: ESM for growth, transmission and virulence [file rstb20160094supp1.pdf]

Supplementary Materials

Growth rate, transmission mode and virulence in human pathogens

Helen C. Leggett, Charlie K. Cornwallis, Angus Buckling & Stuart A. West

Contents:

Fig. S1. Case fatality rate, growth rate and infective dose across the phylogeny of pathogens……………………………………………………………………………………………2

Fig. S2. Virulence and parasite life history ………………………………………………………………… 3

Fig. S3. Growth rates and parasite life history………………………………………………… ……….4

Table S1. MR-BPMM used to calculate the phylogenetic and residual correlations between case fatality rate and pathogen growth rate………………………………………………..5

Table S2. MR-BPMM of case fatality rate and pathogen growth rate across bacterial species…………………………………………………………………………………………………...6

Table S3. MR-BPMM of case fatality rate and pathogen growth rate including life-history and epidemiological factors………………………………………………………………………………7

Table S4. MR-BPMM of case fatality rate and pathogen growth rate including life-history and epidemiological factors across bacterial species ……………………………………………9

Table S5. MR-BPMM of case fatality rate, pathogen growth rate and infective dose including life-history and epidemiological factors……………………………………………………..11

Supplementary R code……………………………………………………………………………… 14

Table S6. Pathogen species and life history data………………………………………………… ..16

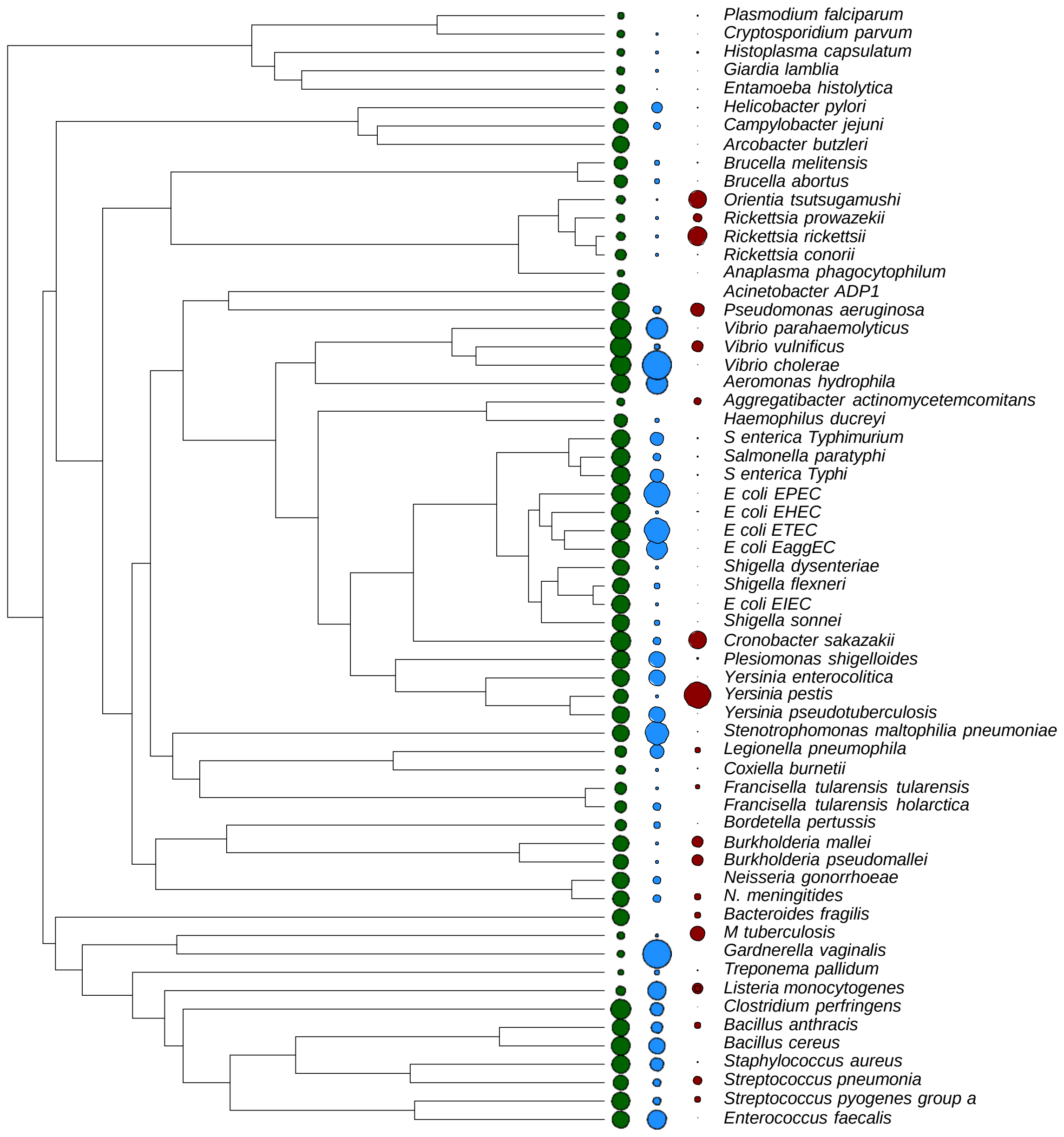

Fig. S1: The pattern of case fatality rate (%: red circles), infective dose (log: blue circles) and generations per week (log: green circles) across the phylogeny. The size of circles represent phenotypic values.

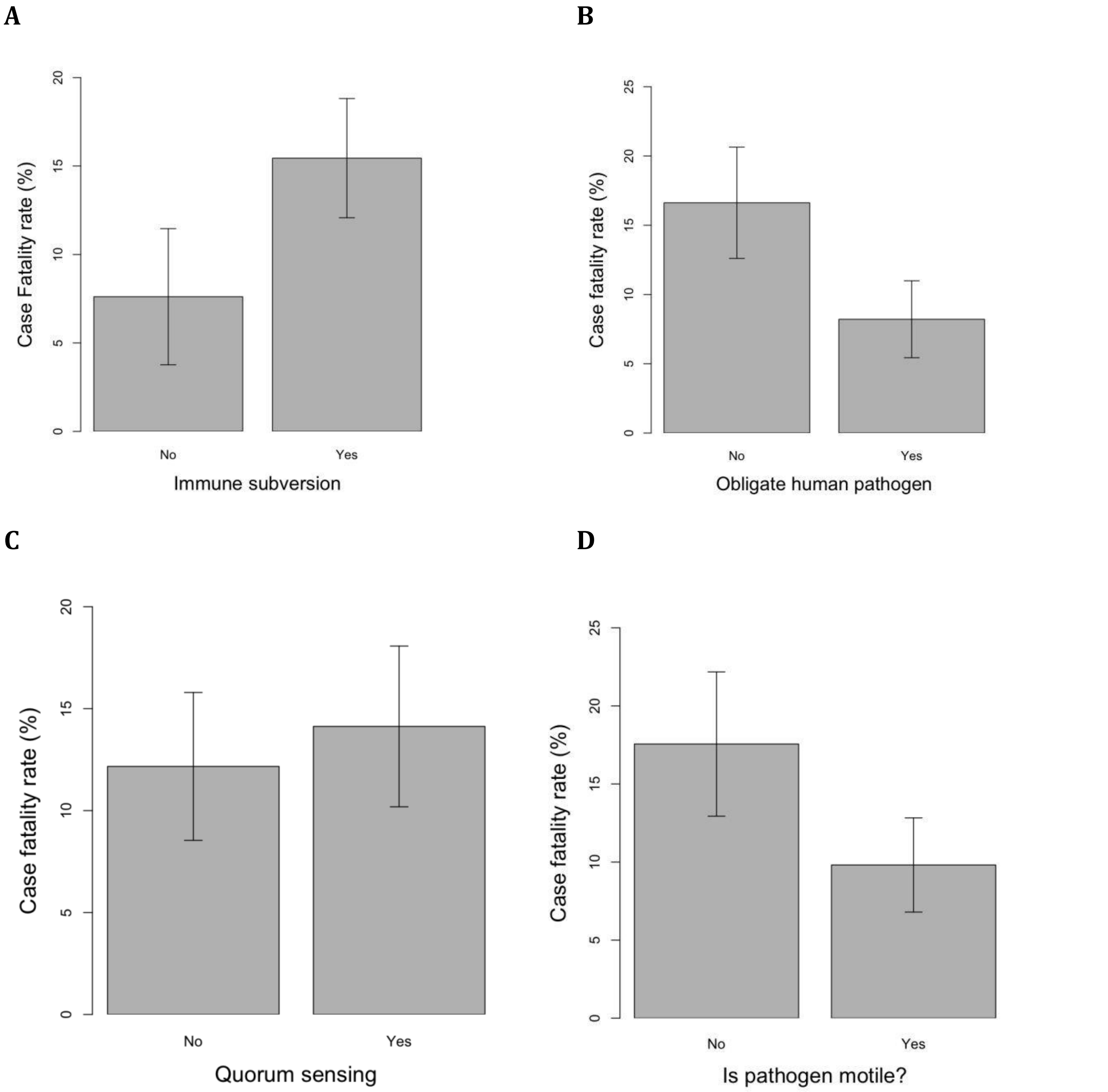

Fig S2. Virulence and parasite life history. The case fatality rate (log number of deaths per 10,000) cases was not correlated with: **(a)** how symptoms of infection affect transmission; **(b)** whether species require a human infection to complete their life cycle (facultative), or not (obligate); **(c)** whether quorum sensing was used to control the production of virulence factors; **(d)** whether species are motile or not (Table S1; S3)

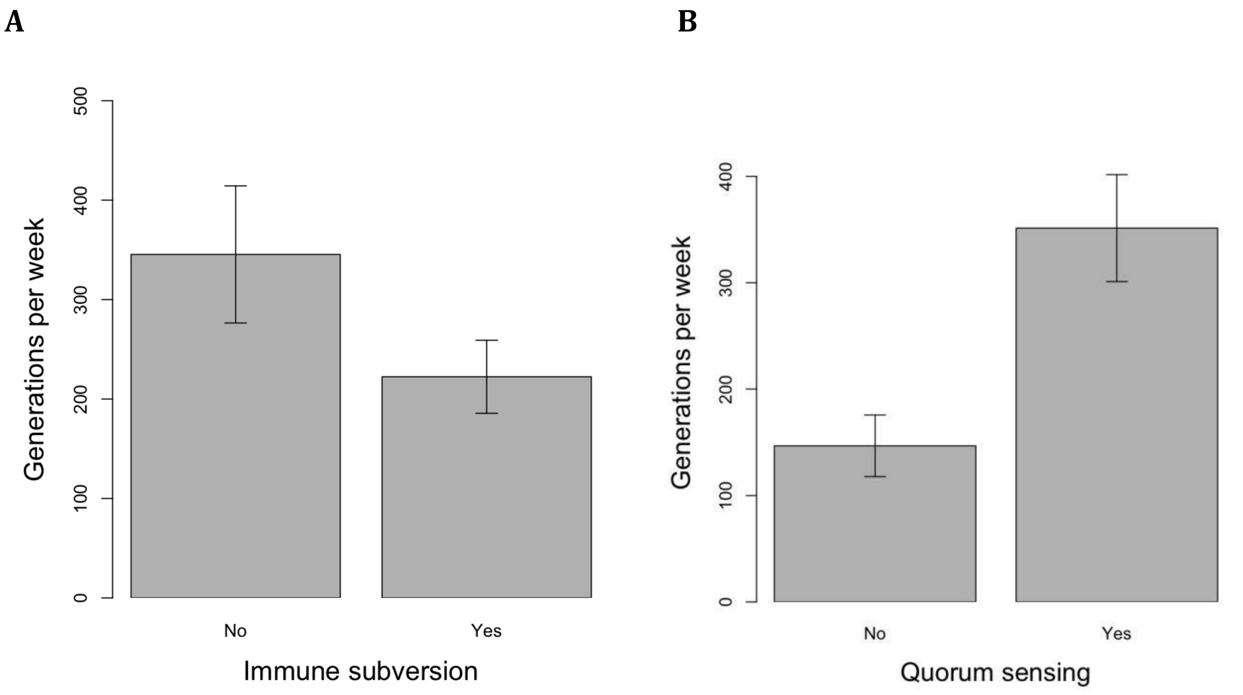

Fig. S3. Growth rates and parasite life history. **(a)** We did not find a significant relationship between immune subversion and generation time (Table S3; S5). **(b)** The generation time of pathogens is significantly lower where quorum sensing was used to control production of virulence factors (Table S3, Table S5).

Table S1. The phylogenetic and residual correlations between case fatality rate (% of cases that result in fatalities: binomial response, logit link function), and the number of generations per week (Poisson response, log link function) across human pathogens estimated using multi-response phylogenetic mixed models. The analysis did not include any other life-history traits.

**Response Traits**	**Random effects**	**Posterior mode (CI)**	**Phylo H^2 (CI)**
Case fatality rate	Phylogenetic variance (V_{a1})	0.02 (0.00, 3.08)	0.14 (0.00, 31.80)
	Residual variance (V_{e1})	7.97 (3.55,11.60)	
Generation time	Phylogenetic variance (V_{a2})	1.54 (1.03, 2.47)	97.48 (91.39, 99.95)
	Residual variance (V_{e2})	0.03 (0.001, 0.15)	
**Correlation**			**P**
Case fatality rate: Generation time	Phylogenetic correlation ($Cov_{a1, a2}$/sqrt($V_{a1} * V_{a2}$))	-0.99 (-0.99, 0.94)	0.43
	Residual correlation ($Cov_{e1, e2}$/sqrt($V_{e1} * V_{e2}$))	-0.99 (-0.99, -0.66)	**0.009**

Table S2. The phylogenetic and residual correlations between case fatality rate (% of cases that result in fatalities: binomial response, logit link function), and the number of generations per week (Poisson response, log link function) across only bacterial human pathogens estimated using multi-response phylogenetic mixed models. The analysis did not include any other life-history traits.

**Response Traits**	**Random effects**	**Posterior mode (CI)**	**Phylo H^2 (CI)**
Case fatality rate	Phylogenetic variance (V_{a1})	0.01 (0.00, 4.78)	0.11 (0.00, 43.35)
	Residual variance (V_{e1})	7.83 (3.68,14.05)	
Generation time	Phylogenetic variance (V_{a2})	1.59 (0.99, 2.45)	98.32 (92.43, 99.95)
	Residual variance (V_{e2})	0.03 (0.001, 0.13)	
**Correlation**			**P**
Case fatality rate: Generation time	Phylogenetic correlation ($Cov_{a1, a2}$/sqrt(V_{a1}* V_{a2}))	-0.99 (-0.99, 0.92)	0.30
	Residual correlation ($Cov_{e1, e2}$/sqrt(V_{e1}* V_{e2}))	-0.99 (-0.99, -0.39)	**0.02**

Table S3: The phylogenetic and residual correlations between case fatality rate (% of cases that result in fatalities: binomial response, logit link function), and the number of generations per week (Poisson response, log link function) across human pathogens estimated using multi-response phylogenetic mixed models with life-history traits included as fixed effects. Fixed effect estimates are on the scale of the link function for each response trait.

Response Trait	Fixed effects	Posterior mode (CI)	pMCMC
Case fatality rate	Immune subversion: No	-3.27 (-5.60, -1.22)	
	Immune subversion: Yes	-2.46 (-4.78, -1.06)	
	Difference	-0.48 (-2.11, 1.11)	0.27
	Infection route: Ingestion	-3.80 (-6.23, -2.07)	
	Infection route: Inhalation	-0.30 (-2.52, 1.56)	
	Infection route: Skin	-1.24 (-3.08, 0.63)	
	Ingestion - inhalation	-3.41 (-5.20, -1.52)	**0.001**
	Ingestion - skin	-2.67 (-4.76, -1.32)	**0.001**
	Inhalation - skin	0.29 (-1.43, 2.49)	0.30
	Symptoms: hinder (1)	-1.78 (-4.72, 0.13)	
	Symptoms: no effect (2)	-2.58 (-4.33, -0.13)	
	Symptoms: help (3)	-3.43 (-5.61, -1.88)	
	Difference 1 - 2	-0.09 (-2.24, 2.23)	0.47
	Difference 1 - 3	0.66 (-0.88, 3.36)	0.13
	Difference 2 - 3	1.24 (-0.23, 3.09)	0.06
	Facultative	-2.50 (-4.99, -0.65)	
	Obligate	-3.89 (-5.77, -1.33)	
	Difference	0.67 (-0.93, 2.22)	0.23
	Quorum Sensing: No	-3.43 (-5.08, -0.76)	
	Quorum Sensing: Yes	-3.29 (-5.37, -1.05)	
	Difference	0.14 (-1.34, 1.63)	0.433
	Pathogen motility: No	-3.28 (-5.32, -1.18)	
	Pathogen motility: Yes	-2.98 (-4.46, -0.27)	
	Difference	-0.28 (-2.13, 0.78)	0.21
Generation Time	Immune subversion: No	3.61 (1.96, 4.60)	
	Immune subversion: Yes	3.53 (2.22, 4.65)	
	Difference	-0.24 (-0.63, 0.32)	0.31
	Infection route: Ingestion	3.31 (1.68, 4.27)	
	Infection route: Inhalation	2.71 (1.38, 4.15)	
	Infection route: Skin	2.90 (1.60, 3.99)	
	Ingestion vs inhalation	0.28 (-0.30, 0.89)	0.11

	Ingestion vs skin	0.18 (-0.32, 0.91)	0.15
	Inhalation vs skin	0.04 (-0.78, 0.71)	0.48
	Symptoms: hinder (1)	2.90 (1.42, 4.63)	
	Symptoms: no effect (2)	3.42 (2.17, 4.40)	
	Symptoms: help (3)	2.79 (1.81, 4.05)	
	Difference 1 - 2	-0.33 (-1.17, 0.85)	0.38
	Difference 1 - 3	0.20 (-1.05, 0.96)	0.46
	Difference 2 - 3	0.01 (-0.31, 0.70)	0.21
	Facultative	3.67 (1.73, 4.48)	
	Obligate	3.47 (1.72, 4.52)	
	Difference	0.18 (-0.50, 0.60)	0.36
	Quorum Sensing: No	3.16 (1.93, 4.71)	
	Quorum Sensing: Yes	3.54 (2.35, 5.08)	
	Difference	-0.45 (-0.99, 0.07)	**0.05**
	Pathogen motility: No	3.44 (1.85, 4.47)	
	Pathogen motility: Yes	3.24 (2.05, 4.75)	
	Difference	-0.27 (-0.72, 0.14)	0.07
	**Random effects**	**Posterior mode (CI)**	**Phylo H^2 (CI)**
Case fatality rate	Phylogenetic variance (V_{a1})	0.01 (0.00, 1.70)	0.08 (0.00, 28.83)
	Residual variance (V_{e1})	3.64 (2.29, 8.43)	
Generation time	Phylogenetic variance (V_{a2})	1.54 (0.95, 2.50)	99.46 (92.77, 99.98)
	Residual variance (V_{e2})	0.01 (0.001, 0.16)	
**Correlation**	**Type of correlation**	**Posterior mode (CI)**	**pMCMC**
Case fatality rate: Generation time	Phylogenetic correlation ($Cov_{a1, a2}$/sqrt(V_{a1}* V_{a2})	0.99 (-0.87, 0.99)	0.26
	Residual correlation ($Cov_{e1, e2}$/sqrt(V_{e1}* V_{e2})	-0.99(-0.99, -0.02)	**0.05**

Table S4: The phylogenetic and residual correlations for bacterial species only between case fatality rate (% of cases that result in fatalities: binomial response, logit link function), and the number of generations per week (Poisson response, log link function) estimated using multi-response phylogenetic mixed models with life-history traits included as fixed effects. Fixed effect estimates are on the scale of the link function for each response trait.

Response Trait	Fixed effects	Posterior mode (CI)	pMCMC
Case fatality rate	Immune subversion: No	-2.98 (-5.20, -0.46)	
	Immune subversion: Yes	-2.17 (-4.27, -0.43)	
	Difference	-0.75 (-2.45, 1.07)	0.29
	Infection route: Ingestion	-3.75 (-5.83, -1.35)	
	Infection route: Inhalation	-0.18 (-2.35, 2.06)	
	Infection route: Skin	-0.57 (-3.10, 1.27)	
	Ingestion - inhalation	-3.70 (-5.69, -1.58)	**0.003**
	Ingestion - skin	-3.17 (-5.00, -0.68)	**0.003**
	Inhalation - skin	0.99 (-1.79, 3.12)	0.30
	Symptoms: hinder (1)	-2.18 (-4.46, 0.75)	
	Symptoms: no effect (2)	-1.18 (-4.22, 0.65)	
	Symptoms: help (3)	-3.50 (-5.10, -0.84)	
	Difference 1 - 2	-0.45 (-3.43, 2.15)	0.36
	Difference 1 - 3	1.47 (-1.51, 3.55)	0.21
	Difference 2 - 3	1.23 (-0.55, 3.52)	0.06
	Facultative	-2.35 (-4.72, -0.13)	
	Obligate	-2.84 (-5.67, -0.93)	
	Difference	0.75 (-0.99, 2.80)	0.20
	Quorum Sensing: No	-2.96 (-4.99, -0.25)	
	Quorum Sensing: Yes	-3.15 (-5.21, -0.65)	
	Difference	0.74 (-1.35, 2.26)	0.36
	Pathogen motility: No	-2.08 (-5.05, -0.50)	
	Pathogen motility: Yes	-2.54 (-4.31, 0.30)	
	Difference	-0.91 (-2.36, 1.08)	0.22
Generation Time	Immune subversion: No	3.83 (2.05, 4.98)	
	Immune subversion: Yes	3.90 (2.38, 5.01)	
	Difference	-0.13 (-0.64, 0.32)	0.25
	Infection route: Ingestion	3.34 (1.81, 4.55)	
	Infection route: Inhalation	3.24 (1.44, 4.25)	
	Infection route: Skin	2.93 (1.67, 4.14)	
	Ingestion vs inhalation	0.44 (-0.24, 0.95)	0.11

	Ingestion vs skin	0.36 (-0.29, 1.06)	0.16
	Inhalation vs skin	0.04 (-0.75, 0.83)	0.48
	Symptoms: hinder (1)	3.37 (1.80, 4.76)	
	Symptoms: no effect (2)	3.73 (2.21, 4.63)	
	Symptoms: help (3)	3.39 (2.13, 4.38)	
	Difference 1 - 2	-0.10 (-1.19, 0.86)	0.43
	Difference 1 - 3	0.48 (-0.80, 1.10)	0.37
	Difference 2 - 3	0.27 (-0.24, 0.80)	0.17
	Facultative	3.52 (2.12, 4.86)	
	Obligate	3.31 (2.00, 4.69)	
	Difference	0.22 (-0.39, 0.80)	0.26
	Quorum Sensing: No	3.73 (2.04, 4.79)	
	Quorum Sensing: Yes	4.02 (2.53, 5.25)	
	Difference	-0.51 (-1.04, 0.06)	**0.03**
	Pathogen motility: No	3.43 (1.87, 4.87)	
	Pathogen motility: Yes	3.47 (2.21, 5.15)	
	Difference	-0.91 (-2.36, 1.08)	0.22
	**Random effects**	**Posterior mode (CI)**	**Phylo H^2 (CI)**
Case fatality rate	Phylogenetic variance (V_{a1})	0.03 (0.00, 2.63)	0.13 (0.00, 35.16)
	Residual variance (V_{e1})	4.99 (2.78,11.41)	
Generation time	Phylogenetic variance (V_{a2})	1.38 (0.76, 2.93)	99.85 (92.98, 99.98)
	Residual variance (V_{e2})	0.002 (0.00, 0.09)	
**Correlation**	**Type of correlation**	**Posterior mode (CI)**	**pMCMC**
Case fatality rate: Generation time	Phylogenetic correlation ($Cov_{a1, a2}$/sqrt($V_{a1} * V_{a2}$))	0.99 (-0.98, 1.00)	0.38
	Residual correlation ($Cov_{e1, e2}$/sqrt($V_{e1} * V_{e2}$))	-0.99(-0.99, 0.64)	0.11

Table S5: The phylogenetic and residual correlations between case fatality rate (% of cases that result in fatalities: binomial response, logit link function), number of generations per week (Poisson response, log link function) and infectious dose (count of parasites: Poisson response, log link function) across human pathogens estimated using multi-response phylogenetic mixed models with life-history traits included as fixed effects. Fixed effect estimates are on the scale of the link function for each response trait.

Response Trait	Fixed effects	Posterior mode (CI)	pMCMC
Case fatality rate	Immune subversion: No	-1.51 (-5.47, 1.79)	
	Immune subversion: Yes	0.29 (-3.10, 3.69)	
	Difference	0.21 (-2.72, 3.29)	0.90
	Infection route: Ingestion	-3.24 (-6.18, -0.45)	
	Infection route: Inhalation	1.08 (-1.17, 4.98)	
	Infection route: Skin	0.23 (-2.95, 3.07)	
	Ingestion vs inhalation	-5.01 (-8.12, -0.90)	**0.002**
	Ingestion vs skin	-2.35 (-6.53, 0.13)	**0.04**
	Inhalation vs skin	1.99 (-1.47, 5.10)	0.16
	Symptoms: hinder (1)	0.43 (-3.51, 3.53)	
	Symptoms: no effect (2)	0.11 (-2.92, 4.10)	
	Symptoms: help (3)	-1.88 (-5.86, 0.76)	
	Difference 1 vs 2	-0.16 (-6.07, 3.19)	0.34
	Difference 1 vs 3	1.68 (-2.20, 7.15)	0.19
	Difference 2 vs 3	4.55 (-1.20, 7.06)	0.08
	Facultative	-0.32 (-4.05, 3.36)	
	Obligate	-0.43 (-4.15, 2.77)	
	Difference	-0.61 (-3.74, 2.02)	0.48
	Quorum Sensing: No	0.58 (-3.28, 3.85)	
	Quorum Sensing: Yes	-2.48 (-5.25, 2.21)	
	Difference	-2.27 (-4.62, 0.83)	0.19
	Pathogen motility: No	0.36 (-3.04, 4.47)	
	Pathogen motility: Yes	0.37 (-4.55, 2.43)	
	Difference	-1.19 (-3.73, 2.04)	0.66
Generation Time	Immune subversion: No	3.16 (1.79, 4.79)	
	Immune subversion: Yes	3.35 (2.07, 4.65)	
	Difference	0.30 (-0.33, 0.74)	0.62
	Infection route: Ingestion	2.83 (1.54, 4.24)	
	Infection route: Inhalation	2.18 (0.91, 3.67)	

	Infection route: Skin	2.37 (1.17, 3.72)	
	Ingestion vs inhalation	0.53 (-0.21, 1.16)	0.11
	Ingestion vs skin	0.41 (-0.19, 1.01)	0.10
	Inhalation vs skin	-0.06 (-0.84, 0.73)	0.45
	Symptoms: hinder (1)	2.73 (1.18, 4.22)	
	Symptoms: no effect (2)	3.07 (1.68, 3.97)	
	Symptoms: help (3)	2.72 (1.39, 3.57)	
	Difference 1 vs 2	0.03 (-1.03, 1.02)	0.45
	Difference 1 vs 3	0.35 (-0.89, 1.14)	0.37
	Difference 2 vs 3	0.34 (-0.26, 0.75)	0.15
	Facultative	3.21 (1.38, 4.28)	
	Obligate	2.60 (1.18, 4.19)	
	Difference	-0.15 (-0.57, 0.49)	0.48
	Quorum Sensing: No	2.68 (1.13, 4.06)	
	Quorum Sensing: Yes	2.91 (1.66, 4.59)	
	Difference	0.36 (-0.06, 1.03)	0.06
	Pathogen motility: No	3.13 (1.28, 4.17)	
	Pathogen motility: Yes	2.99 (1.76, 4.54)	
	Difference	0.30 (-0.12, 0.80)	0.14
Infective dose	Immune subversion: No	4.95 (1.52, 7.68)	
	Immune subversion: Yes	0.02 (-3.43, 1.93)	
	Difference	-4.53 (-6.67, -1.91)	**<0.001**
	Infection route: Ingestion	2.19 (0.14, 5.48)	
	Infection route: Inhalation	0.18 (-2.65, 3.29)	
	Infection route: Skin	1.77 (-1.35, 4.67)	
	Ingestion vs inhalation	2.36 (-0.89, 5.85)	0.10
	Ingestion vs skin	2.18 (-1.99, 4.27)	0.25
	Inhalation vs skin	-1.50 (-4.89, 2.14)	0.24
	Symptoms: hinder (1)	0.62 (-2.38, 3.99)	
	Symptoms: no effect (2)	2.76 (-0.94, 5.04)	
	Symptoms: help (3)	2.41 (-1.09, 4.54)	
	Difference 1 vs 2	-1.25 (-5.19, 2.70)	0.24
	Difference 1 vs 3	-0.11 (-4.66, 2.81)	0.31
	Difference 2 vs 3	-0.28 (-2.36, 3.38)	0.41
	Facultative	1.67 (-0.73, 4.84)	
	Obligate	2.62 (-0.35, 5.38)	
	Difference	1.19 (-0.69, 4.02)	0.21
	Quorum Sensing: No	0.46 (-1.84, 3.61)	
	Quorum Sensing: Yes	4.04 (1.21, 6.79)	

	Difference	3.38 (0.94, 5.61)	**0.008**
	Pathogen motility: No	2.48 (-1.37, 4.13)	
	Pathogen motility: Yes	2.85 (0.43,5.98)	
	Difference	2.28 (-0.37, 4.21)	0.11
**Response Traits**	**Type of correlation**	**Posterior mode (CI)**	**pMCMC**
Case fatality rate: Generation time	Phylogenetic correlation ($Cov_{a1, a2}$/sqrt(V_{a1}* V_{a2})	-0.99 (-0.99, 0.98)	0.30
	Residual correlation ($Cov_{e1, e2}$/sqrt(V_{e1}* V_{e2})	0.13 (-0.99, 0.70)	0.37
Case fatality rate: Infective dose	Phylogenetic correlation ($Cov_{a1, a3}$/sqrt(V_{a1}* V_{a3})	-0.99 (-0.99, 0.98)	0.98
	Residual correlation ($Cov_{e1, e3}$/sqrt(V_{e1}* V_{e3})	-0.58 (-0.78, -0.23)	**0.001**
Generation time: Infective dose	Phylogenetic correlation ($Cov_{a2, a3}$/sqrt(V_{a2}* V_{a3})	0.99 (0.16, 0.99)	**0.02**
	Residual correlation ($Cov_{e2, e3}$/sqrt(V_{e2}* V_{e3})	-0.96 (-0.99, 0.80)	0.24
**Response Trait**	**Random effects**	**Posterior mode (CI)**	**Phylo H^2 (CI)**
Case fatality rate	Phylogenetic variance (V_{a1})	0.10 (0.000, 14.68)	0.14 (0.000, 21.58)
	Residual variance (V_{e1})	52.05 (33.29, 88.13)	
Generation time	Phylogenetic variance (V_{a2})	1.54 (0.95, 2.50)	99.50 (90.78, 99.97)
	Residual variance (V_{e2})	0.01 (0.001, 0.16)	
Infective dose	Phylogenetic variance (V_{a3})	3.48 (0.02, 28.52)	17.86 (0.10, 67.96)
	Residual variance (V_{e3})	17.66 (9.21, 33.01)	

Supplementary R code

Priors that were tested for MR-BPMMs

MR_Prior1 = list(R = list(V = diag(2), nu=0.002), G = list(G1=list(V = diag(2)*(0.002/1.002), nu =1.002))

MR_Prior2= list(G=list(G1=list(V=diag(2),n=0.002, alpha.mu=rep(0,2), alpha.V=diag(2)*1000)), R=list(V=diag(2),n=0.002))

MR_Prior3 = list(R = list(V = diag(3), nu=0.002), G = list(G1=list(V = diag(3)*(0.002/2.002), nu =2.002))

MR_Prior4=list(R=list(V=diag(3),n=0.002), G=list(G1=list(V=diag(3),n=1, alpha.mu=rep(0,3), alpha.V=diag(3)*1000)))

R code for Table S1

MCMCglmm(cbind(cbind(%_dead, %_alive), generations_week) ~ trait-1, random = ~us(trait):animal, rcov = ~us(trait):units, pedigree=tree, family =c("multinomial2","poisson"), nodes="ALL", data = data, prior=MR_Prior2, nitt=10000000, burnin=5000000, thin=5000)

R code for Table S2

MCMCglmm(cbind(cbind(%_dead, %_alive), generations_week) ~ trait-1, random = ~us(trait):animal, rcov = ~us(trait):units, pedigree=tree, family =c("multinomial2","poisson"), nodes="ALL", data = bacteria_data, prior=MR_Prior2, nitt=10000000, burnin=5000000, thin=5000)

R code for Table S3

MCMCglmm(cbind(cbind(%_dead, %_alive), generations_week, Infectious_dose) ~ trait-1 + trait:immune_subversion + trait:infection_route + trait:symptoms + trait:obligate_pathogen + trait:quorum_sensing + trait:motility, random = ~us(trait):animal, rcov = ~us(trait):units, pedigree=tree, family =c("multinomial2","poisson"), nodes="ALL", data = data, prior=MR_Prior2, nitt=10000000, burnin=5000000, thin=5000)

R code for Table S4

MCMCglmm(cbind(cbind(%_dead, %_alive), generations_week, Infectious_dose) ~ trait-1 + trait:immune_subversion + trait:infection_route + trait:symptoms + trait:obligate_pathogen + trait:quorum_sensing + trait:motility, random = ~us(trait):animal, rcov = ~us(trait):units,

pedigree=tree, family =c("multinomial2","poisson","poisson"), nodes="ALL", data = data, prior=MR_Prior4, nitt=10000000, burnin=5000000, thin=5000)

Table S6: Pathogen species and life history data

Species	Obligate or facultative human parasitism	Immune subversion	Generation time (hr)	Case fatality rate (%)	QS- reg. virulence	Infection route	Effect of symptoms of infection on transmission	Motility	Infective dose	Source
Acinetobacter ADP1	Facultative	0	0.5	?	0	Skin	No effect	0	?	[1-4]
Actinobacillus actinomycetemco-mitans	Facultative	1	11	25.5	0	Skin	No effect	0	?	[5-9]
Aeromonas hydrophila	Facultative	0	0.35	?	1	Ingestion	Helps	1	50,500,000	[10-13]
Anaplasma phagocytophilum	Facultative	1	21.6	1.5	0	Skin	Hinders	?	?	[1,14-16]
Arcobacter butzleri	Facultative	?	0.66	0.4	0	Ingestion	Helps	1	?	[1,14-17]
Bacillus anthracis	Facultative	1	0.5	20	1	Skin	Helps	0	20,250	[1,10, 18]
Bacillus cereus	Facultative	0	0.3	0	1	Ingestion	Helps	1	1,000,000	[1,10, 18]
Bacteroides fragilis	Facultative	1	0.63	19.3	0	Skin	No effect	0	?	[2,19
Bordetella	Obligate	0	3.8	1	0	Inhalation	Helps	0	200	[1,20,

pertussis										21]
Brucella abortus	Facultative	1	2	1.025	1	Ingestion	No effect	0	55	[18,22-25]
Brucella melitensis	Facultative	1	2	3.25	1	Ingestion	No effect	0	55	[1,10, 18,22-24]
Burkholderia pseudomallei	Facultative	1	1	38.5	1	Inhalation	No effect	1	10	[10,25, 26]
Burkholderia mallei	Facultative	1	0.75	38.5	1	Inhalation	No effect	0	10	[1,10, 25,26]
Campylobacter jejuni	Facultative	1	1.1	0.4	0	Ingestion	Helps	1	550	[1,10, 18
Clostridium perfringens	Facultative	1	0.2	0.07	1	Ingestion	Helps	1	100,000	[1,10, 18]
Coxiella burnetii	Facultative	1	8	3	0	Inhalation	No effect	0	10	[1,10, 18]
Cronobacter sakazakii	Facultative	1	0.23	60	1	Skin	Hinders	1	1000	[1,27-29]
Cryptosporidium parvum	Facultative	1	13	0.5	0	Ingestion	Helps	1	5.5	[18,30, 31]
Escherichia coli	Obligate	0	0.5	0.025	1	Ingestion	Helps	1	50,500,000	[10,18]

EaggEC										
Escherichia coli EHEC	Obligate	1	0.33	0.254	1	Ingestion	Helps	1	10	[10,18]
Escherichia coli EIEC	Obligate	1	0.4	0.025	0	Ingestion	Helps	1	10	[10,18]
Escherichia coli EPEC	Obligate	0	0.4	0.025	1	Ingestion	Helps	1	3.37E+09	[10,18]
Escherichia coli ETEC	Obligate	0	0.33	0.025	1	Ingestion	Helps	1	2.55E+09	[10,18]
Entamoeba histolytica	Facultative	1	9.6	2	1	Ingestion	Helps	1	1	[18,32, 33]
Enterococcus faecalis	Facultative	0	0.5	0.01	1	Ingestion	Helps	0	10,000,000	[1,10, 34]
Francisella tularensis, holarctica	Facultative	1	3	0	1	Ingestion	Hinders	0	1000	[1,18]
Francisella tularensis, tularensis	Facultative	1	3	14	1	Inhalation	Hinders	0	7.5	[1,10, 18]
Gardnerella vaginalis	Facultative	0	13.5	0	1	Skin	Helps	0	2.00E+10	[10,34]

Giardia lamblia	Facultative	0	10.5	0.6	0	Ingestion	Helps	1	10	[18,34]
Haemophilus ducreyi	Obligate	1	1.8	0	0	Skin	Helps	1	30	[1,10]
Helicobacter pylori	Obligate	1	2.4	3	0	Ingestion	Helps	1	10,000	[1-3, 19]
Histoplasma capsulatum	Facultative	1	13	6	1	Inhalation	No effect	0	10	[1,18, 35-37]
Legionella pneumophila	Obligate	1	3.3	17.5	1	Inhalation	No effect	1	140,000	[10,18]
Listeria monocytogenes	Obligate	1	1	30	1	Ingestion	No effect	1	1000	[1,10, 18]
Mycobacterium tuberculosis	Obligate	1	19	50	0	Inhalation	Helps	0	10	[10,18 38]
Mycoplasma pneumonia	Obligate	0	6	36.3	0	Inhalation	Helps	1	5,050,000	[1,10, 39]
Neisseria meningitides	Obligate	0	0.72	20.3	0	Inhalation	No effect	0	1000	[1,10, 14]
Neisseria gonorrhoeae	Obligate	1	0.58	0	0	Skin	Hinders	0	1050	[1,10, 18]
Orientia tsutsugamushi	Facultative	1	9	60	0	Skin	Hinders	0	3	[10,40]

Plasmodium falciparum	Obligate	1	24	4	0	Skin	Helps	1	?	[41-43]
Plesiomonas shigelloides	Facultative	0	0.43	6	0	Ingestion	Helps	1	1,000,000	[10,44]
Pseudomonas aeruginosa	Facultative	0	0.5	47	1	Skin	Helps	1	1000	[1,10, 18]
Rickettsia conorii	Obligate	1	4.1	2.1	0	Skin	Hinders	0	10	[1,18, 40]
Rickettsia prowazekii	Obligate	1	10	30	0	Skin	Hinders	0	10	[1,10, 18]
Rickettsia rickettsia	Obligate	1	9	65	0	Skin	Hinders	0	10	[1,10, 18,45]
Salmonella enterica Typhi	Obligate	1	0.4	4	0	Ingestion	No effect	1	100,000	[10]
Salmonella enterica Typhimurium	Facultative	1	0.4	4	1	Ingestion	Helps	1	100,000	[1,10, 18]
Salmonella paratyphi	Obligate	1	0.4	4	0	Ingestion	No effect	1	1000	[1,18]
Shigella dysenteriae	Obligate	1	0.67	0.6	0	Ingestion	Helps	0	10	1,46]

Shigella flexneri	Obligate	1	0.68	0.1	0	Ingestion	Helps	0	100	[2,18]
Shigella sonnei	Obligate	1	0.5	0.9	0	Ingestion	Helps	0	75	[1,47]
Staphylococcus aureus	Facultative	1	0.4	4	1	Ingestion	No effect	0	100,000	[1,10, 18]
Stenotrophomonas maltophilia pneumoniae	Obligate	0	0.6	?	1	Ingestion	No effect	1	5.01E+08	[1,10]
Streptococcus pyogenes	Obligate	1	0.4	19	1	Inhalation	No effect	0	1000	[1,10, 18]
Treponema pallidum	Obligate	1	33	3.17	0	Skin	Helps	1	57	[1,10 48]
Vibrio cholera	Obligate	0	0.2	0.9	1	Ingestion	Helps	1	3.33E+10	[1,10, 18,34]
Vibrio parahaemolyticus	Facultative	0	0.2	0.01	1	Ingestion	No effect	1	55,000,000	[1,10, 34]
Vibrio vulnificus	Facultative	1	0.16	39	1	Ingestion	Helps	1	100	[1,10, 34]
Yersinia enterocolitica	Facultative	1	0.55	0.5	1	Ingestion	Helps	1	1,000,000	[10,18]
Yersinia pseudotuberculosis	Facultative	1	0.5	0.5	1	Ingestion	No effect	0	1,000,000	[1,10, 34]

Yersinia pestis	Facultative	1	1.25	90	1	Skin	Helps	0	10	[1,10, 18]

Table S7 references

1. Vieira-Silva S & Rocha EPC (2010) The systemic imprint of growth and its uses in ecological (mata)genomics. PLoS Genetics 6: e1000808
2. Chastre, J & Trouillet JL (2000) Problem pathogens (Pseudomonas aeruginosa and Acinetobacter). Semin Respir Infect 15, 287-298
3. Qiu H *et al.* (2012) Role of Macrophages in Early Host Resistance to Respiratory Acinetobacter baumannii Infection. PLoS ONE 7, e40019
4. Bhargava N *et al.* (2010) Quorum sensing in Acinetobacter: an emerging pathogen. Critical Reviews in Microbiology 36, 349-360
5. Paturel L *et al.* (2004) Actinobacillus actinomycetemcomitans endocarditis. Clin Microbiol Infect 10, 98-118
6. Yogev R *et al.* (1986) In vitro activity of antibiotics alone and in combination against Actinobacillus actinomycetemcomitans. Antimicrob Agents Chemother 29, 179-181
7. Henderson B *et al.* (2002) Actinobacillus actinomycetemcomitans. J Med Microbiol 51, 1013-1020
8. Silva MT (2010) Bacteria-induced phagocyte secondary necrosis as a pathogenicity mechanism. J Leukoc Biol 88, 885-896
9. Fong KP *et al.* (2003) luxS and arcB control aerobic growth of Actinobacillus actinomycetemcomitans under iron limitation. Infect Immun 71, 298-308
10. Gama JOA *et al.* (2012) Immune Subversion and Quorum-Sensing Shape the Variation in Infectious Dose among Bacterial Pathogens. PLoS Pathog 8, e1002503
11. Okumura K *et al.* (2011) Severe sepsis caused by Aeromonas hydrophila in a patient using tocilizumab: a case report. J Med Case Rep 5, 1752-1947

12. Loghothetis PN & Austin B (1996) Variations in antigenicity of Aeromonas hydrophila strains in rainbow trout (Oncorhynchus mykiss, Walbaum): an association with surface characteristics. Fish Shellfish Immunol 23, 47-55

13. United States Food and Drug Administration (2003) The Bad Bug Book. Available: http://www.fda.gov/food/foodsafety/foodborneillness/foodborneillnessfoodbornepathogensnaturaltoxins/badbugbook/default.htm.

14. Centers for Disease Control and Prevention. Alphabetical Index of Parasitic Diseases. Available: http://www.cdc.gov/parasites/az/index.html.

15. Garcia-Garcia JC *et al.* (2009) Epigenetic Silencing of Host Cell Defense Genes Enhances Intracellular Survival of the Rickettsial Pathogen *Anaplasma phagocytophilum*. PLoS Pathog 5, e1000488

16. Rikihisa Y (2011) Mechanisms of Obligatory Intracellular Infection with Anaplasma phagocytophilum. Clin. Microbiol. Rev. 24, 469-489

17. Vandenberg O *et al.* (2004) Arcobacter species in humans. Emerg Infect Dis 10, 1863-1867

18. Leggett HC *et al.* (2012) Mechanisms of Pathogenesis, Infective Dose and Virulence in Human Parasites. PLoS Pathog 8, e1002512

19. Redondo MC *et al.* (1995) Attributable mortality of bacteremia associated with the Bacteroides fragilis group. Clin Infect Dis 20, 1492-1496

20. Wilson M, McNab R, Henderson B (2002) *Bacterial Disease Mechanisms: An introduction to cellular microbiology*. Cambridge University Press

21. Mishra M *et al.* (2005) The BvgAS signal transduction system regulates biofilm development in Bordetella. J Bacteriol 187, 1474-1484

22. Moreno E & Moriyón I (2002) Brucella melitensis: A nasty bug with hidden credentials for virulence. Proc Natl Acad Sci USA 99, 1-3

23. Starr T *et al.* (2011) Selective subversion of autophagy complexes facilitates completion of the Brucella intracellular cycle. Cell Host Microbe 11, 33-45

24. Rambow-Larsen AA *et al.* (2008) Putative Quorum-Sensing Regulator BlxR of Brucella melitensis Regulates Virulence Factors Including the Type IV Secretion System and Flagella. J. Bacteriol. 190, 3274-3282

25. Centre for Food Security and Public Health. Available: http://www.cfsph.iastate.edu/Factsheets/pdfs/melioidosis.pdf

26. White NJ (2003) Melioidosis. The Lancet 361, 1715-1722

27. Bowen AB & Braden CR (2006) Invasive Enterobacter sakazakii disease in infants. Emerg Infect Dis 12, 1185-1189

28. Lehner A *et al.* (2005) Biofilm Formation, Extracellular Polysaccharide Production, and Cell-to-Cell Signaling in Various Enterobacter sakazakii Strains: Aspects Promoting Environmental Persistence. J Food Protection 68, 2287-2294

29. Health Canada (2003) Pathogen safety data sheets. Available: http://www.phac-aspc.gc.ca/lab-bio/res/psds-ftss/index-eng.php

30. Plattner F & Soldati-Favre D (2008) Hijacking of Host Cellular Functions by the Apicomplexa. In Ann Rev Microbiol 471-487

31. Water Research Centre. Available: http://www.water-research.net/cryptosporidium.htm

32. Eichinger D (2001) Encystation in parasitic protozoa. Curr Opin Microbiol 4, 421-426

33. Penuliar GM *et al.* (2011) Mechanism of trifluoromethionine resistance in Entamoeba histolytica. J Antimicrob Chemother 66, 2045-2052

34. Medscape. Infectious Disease Articles. Available: http://emedicine.medscape.com/infectious_diseases.

35. Medoff G *et al.* (1986) Correlation between pathogenicity and temperature sensitivity in different strains of Histoplasma-capsulatum. J Clin Invest 78, 1638-1647

36. Youseff BH *et al.* (2012) Extracellular superoxide dismutase protects Histoplasma yeast cells from host-derived oxidative stress. PLoS Pathog 8, 17

37. Strauss E (1999) A Symphony of Bacterial Voices. Science 284, 1302-1304

38. Kramnik I *et al.* (2000) Genetic control of resistance to experimental infection with virulent Mycobacterium tuberculosis. Proc Natl Acad Sci USA 97, 8560-8565

39. O'Brien KL *et al.* (2009) Burden of disease caused by Streptococcus pneumoniae in children younger than 5 years: global estimates. The Lancet 374, 893-902

40. Stramer SL *et al.* (2009) Emerging infectious disease agents and their potential threat to transfusion safety. Transfusion 49, 1S-29S
41. Greenberg AE & Lobel HO (1990) Mortality from Plasmodium falciparum Malaria in Travelers from the United States, 1959 to 1987. Annals Internal Medicine 113, 326-327
42. Olliaro P (2008) Mortality Associated with Severe Plasmodium falciparum Malaria Increases with Age. Clin Infect Dis 47, 158-160
43. Mutai BK & Waitumbi JN (2010) Apoptosis stalks Plasmodium falciparum maintained in continuous culture condition. Malar J S3-S6
44. The Johns Hopkins Medical Institutions, department of pathology: http://pathology5.pathology.jhmi.edu/micro/v15n28.htm
45. Radulovic S *et al.* (2002) Rickettsia-macrophage interactions: host cell responses to Rickettsia akari and Rickettsia typhi. Infect Immun 70, 2576-2582
46. Guerin PJ *et al.* (2003) Shigella dysenteriae serotype 1 in West Africa: intervention strategy for an outbreak in Sierra Leone. The Lancet 362, 705-706
47. Kotloff KL *et al.* (1999) Global burden of Shigella infections: implications for vaccine development and implementation of control strategies. Bull World Health Organ 77, 651-666
48. Garcia-Garcia L *et al.* (2013) Epidemiology of syphilis-related hospitalisations in Spain between 1997 and 2006: a retrospective study. BMJ open 1, e000270
